# The SNP rs6859 in *NECTIN2* gene is associated with underlying heterogeneous trajectories of cognitive changes in older adults

**DOI:** 10.1186/s12883-024-03577-4

**Published:** 2024-02-27

**Authors:** Aravind Lathika Rajendrakumar, Konstantin G. Arbeev, Olivia Bagley, Anatoliy I. Yashin, Svetlana Ukraintseva, for the Alzheimer’s Disease Neuroimaging Initiative

**Affiliations:** Biodemography of Aging Research Unit, Social Science Research Institute, Duke University, Durham, NC, 27708-0408 USA

**Keywords:** MMSE, *NECTIN2*, rs6859, Heterogeneity, Trajectory analysis, Latent class

## Abstract

**Background:**

Functional decline associated with dementia, including in Alzheimer’s disease (AD), is not uniform across individuals, and respective heterogeneity is not yet fully explained. Such heterogeneity may in part be related to genetic variability among individuals. In this study, we investigated whether the SNP rs6859 in nectin cell adhesion molecule 2 *(NECTIN2)* gene (a major risk factor for AD) influences trajectories of cognitive decline in older participants from the Alzheimer’s Disease Neuroimaging Initiative (ADNI).

**Methods:**

We retrospectively analyzed records on 1310 participants from the ADNI database for the multivariate analysis. We used longitudinal measures of Mini-Mental State Examination (MMSE) scores in participants, who were cognitively normal, or having AD, or other cognitive deficits to investigate the trajectories of cognitive changes. Multiple linear regression, linear mixed models and latent class analyses were conducted to investigate the association of the SNP rs6859 with MMSE.

**Results:**

The regression coefficient per one allele dose of the SNP rs6859 was independently associated with MMSE in both cross-sectional (-2.23, *p* < 0.01) and linear mixed models (-2.26, *p* < 0.01) analyses. The latent class model with three distinct subgroups (class 1: stable and gradual decline, class 2: intermediate and late decline, and class 3: lowest and irregular) performed best in the posterior classification, 42.67% (*n* = 559), 21.45% (*n* = 281), 35.88% (*n* = 470) were classified as class 1, class 2, and class 3. In the heterogeneous linear mixed model, the regression coefficient per one allele dose of rs6859 – A risk allele was significantly associated with MMSE class 1 and class 2 memberships and related decline; Class 1 (-2.28, 95% CI: -4.05, -0.50, *p* < 0.05), Class 2 (-5.56, 95% CI: -9.61, -1.51, *p* < 0.01) and Class 3 (-0.37, 95% CI: -1.62, 0.87, *p* = 0.55).

**Conclusions:**

This study found statistical evidence supporting the classification of three latent subclass groups representing complex MMSE trajectories in the ADNI cohort. The SNP rs6859 can be suggested as a candidate genetic predictor of variation in modeling MMSE trajectory, as well as for identifying latent classes with higher baseline MMSE. Functional studies may help further elucidate this relationship.

**Supplementary Information:**

The online version contains supplementary material available at 10.1186/s12883-024-03577-4.

## Background

Dementia is a condition associated with progressive neurodegeneration and subsequent cognitive impairment in older adults [1, 2]. The Mini-Mental State Examination (MMSE) is one of the most popular neuropsychological tests that can be easily administered to measure cognitive changes [3]. Clinical signs of dementia are not obvious at inception and take years to fully manifest [4, 5]. Cognitive decline occurs at a non-uniform rate across age ranges, and this decline can start as early as 29 years in the general population [6]. However, the individual differences in cognitive decline and the factors contributing to these differences remain understudied [7]. For example, only a fraction of the MMSE change was explained by predictors such as age and education in statistical models [6, 8, 9]. A large part of the remaining variation could be explained by genetics [10]. 

Individuals exposed to certain infectious diseases have a higher risk of developing cognitive problems [11]. Among the most common of these are the members of the herpes virus family, whose presence has been linked to the symptoms of Alzheimer’s disease, the most common type of dementia [12, 13]. Importantly, this risk is amplified by genetic variations such as those in *APOE4* and therefore we hypothesize that the same is likely in polymorphisms in genes that facilitate cell to cell spread of infections [14, 15]. The single nucleotide polymorphisms (SNPs) in *NECTIN2* (nectin cell adhesion molecule 2) gene may have implications for cognition due to role of this gene in modulating the entry of herpes viruses into the brain and in AD [15–19]. Despite the strong association between *NECTIN2* gene and AD [19, 20], the exact biological mechanisms behind the association between *NECTIN2* gene and cognitive decline remain unknown [21]. Furthermore, *NECTIN2* gene is located in a major dementia locus along with *TOMM40* and *APOE* genes. Therefore, it is challenging to quantify the actual relationship between the polymorphisms in this gene and AD [22]. 

The *NECTIN2* gene is not in linkage disequilibrium with the *APOE4* variant, which is the rationale behind conducting this investigation. The statistical relationship between rs6859 and MMSE trajectories has not yet been reported [19–21] Herein, we (1) investigate whether there is a statistically significant association for SNP rs6859 with the lowest recorded individual MMSE measurements, (2) identify the trajectory and heterogeneous clusters of MMSE trajectories, and (3) explore association of SNP rs6859 with the identified latent subgroup trajectories in the Alzheimer’s Disease Neuroimaging Initiative (ADNI) database.

## Methods

### Dataset

We used the ADNIMERGE data subset from the Alzheimer’s Disease Neuroimaging Initiative (ADNI) consortium (adni.loni.usc.edu). ADNI is an ongoing longitudinal study intended to gather relevant data on AD. The study began in 2003 under Dr. Michael W. Weiner [23]. The starting goal of the study was to gather insights into the transition of mild cognitive impairment (MCI) and mild Alzheimer’s disease (AD) to more severe forms of dementia by combining information from a plethora of sources, such as imaging, biomarkers, and clinical and cognitive assessments. Participants between 55 and 90 years of age are recruited in different waves (ADNI1, ADNI-GO, ADNI2, and ADNI3). During the participant visit, a wide variety of biomarker assays, including blood and cerebrospinal fluid (CSF), diagnostic brain images, and various cognitive tests, are collected as per standard protocols [24]. The datasets are regularly updated and made publicly accessible to researchers after quality assessments (http://adni.loni.usc.edu/data-samples/access-data/). ADNIMERGE is a subset of ADNI data that links participant information across ADNI datasets from multiple visits, thus making it convenient to perform multivariate analysis. ADNI used multiple genotyping arrays in each wave. For genome-wide association studies (GWAS), Human610-Quad BeadChip, Illumina HumanOmniExpress BeadChip, and the Illumina Infinium Global Screening Array v2 (GSA2) were used in chronological order. The genotype data is stored as PLINK [25, 26] binary files, and the genotype imputation and allele information are available in SNP summary files for each chromosome. The analysis was performed in a mixed sample of cognitively normal, AD, and other individuals with varying degrees of cognitive impairments. We did not treat patients with normal, mildly impaired, and clinical overt dementia separately in the study because our goal was to determine whether rs6859 can predict MMSE trajectories. In this cohort, MMSE assessments take place every six months after the baseline. The MMSE values of all the samples were included as a continuous response variable in the regression models. To facilitate longitudinal analysis, we only included participants with at least two MMSE records in this study [6]. 

### Statistical analysis

The MMSE has a typically skewed distribution, and its main drawbacks include a lesser sensitivity to detect cognitive changes and presence of ceiling/floor effects that violate standard mixed model assumptions [27]. The ceiling/floor effects prevent proper assessment of the cognitive abilities of the individuals under examination [28]. Also, the non-normal distribution of data in the mixture models tends to produce a greater number of unreal latent classes [29]. We therefore used the *NormPsy* package proposed by Philipps et al. to correct for the above-mentioned issues [30]. The package was specifically designed for normalization of the MMSE and other psychometric scales that tend to have a skewed distribution. The normalization henceforth will be referred to as MMSE (normalized). In brief, the transformed values were generated as averaged values using cross validations, comparing and contrasting estimates from latent process and linear mixed models. The transformations allow a better spread of values from the original scale of 0–30 to 0-100, thereby improving the curvilinearity and sensitivity to detect a more biologically meaningful change [31]. 

R software was used for all of the analysis [32]. In general, for all the multivariate regression models, we used the dataset without any missing covariates. A cross-sectional study was conducted to investigate whether SNP rs6859 is associated with the normalized MMSE, for which we extracted the lowest MMSE value for each participant from the longitudinal dataset. We first employed a standard linear regression model with normalized MMSE as the response and SNP rs6859 as the exposure variables. The impact of age, sex, years of education, race, and history of smoking (yes/no) and alcoholism (yes/no) were also controlled in the analysis. We further ascertained the baseline medication use for diabetes, hypertension, dyslipidemia and cardiovascular diseases (CVD) using the medications dataset using the Anatomical Therapeutic Chemical Code (ATC). We first ran the *dredge* function from the *MuMin *package, which uses an AIC criterion for model ranking. Several model subsets were created using this function, which considers ‘all possible’ combinations of the model terms. Following this, we calculated estimates using the model-averaged estimates using the *model.avg* function from the same package. For averaging, we used only the models with AIC scores (delta < 2). The delta AIC is the difference in the AIC scores calculated for the best model and all other model subsets. This type of model averaging is a kind of shrinkage method where weak associations are penalized to zero and considers all variables in all subsets of models [33]. The E-values were used to quantify the effect of residual confounding on the estimates derived from the linear regression analysis. We used the EValue package to compute the E-values for the rs6859 association from the univariate and multivariate regression models with normalized MMSE outcome [34]. E-value estimates provide information regarding the effect size an unmeasured confounder should possess to nullify the observed exposure-treatment outcome conditional on the covariate-adjusted effects. A larger E-value implies that a stronger confounding effect is required to rule out the exposure effect in the observational studies [34]. 

We used the *lmer* function from the lme4 package for performing the linear mixed models to describe the long-term change in MMSE and investigated whether the A allele variation of rs6859 could quantify this change [35]. Age, education, race, sex, marriage status, measurement visits, smoking, and alcohol history were the other model covariates for estimating the fixed effects. Age and education were scaled and centred before being included in the models. In the mixed models, the impact of drugs on the MMSE is represented by modeling the corresponding clinical conditions, which were identified from the medication file. The best set of model covariates was determined using the *anova* function and the maximum likelihood method. The estimates for the best covariate model were computed with the restricted maximum likelihood method. The outcome may be influenced by individual variation and measurement visits. Hence, for the random effects part, the optimal model had varying slopes for clinical visits and random intercepts for the participants. We used the Nelder-Mead optimizer to ensure model convergence [36]. We used the *glmm.hp* function, which decomposes the model variance by employing hierarchical partitioning to identify individual variable contributions (marginal *R*^*2*^) to the model [37]. 

Next, we ran latent class models to investigate the predictive utility of SNP rs6859. Latent class models are a kind of modified finite mixture model used for the data where the distribution of the response variable (y) is conditionally distributed across K latent classes [38]. It is not possible to infer the number of latent classes underlying the response variable beforehand. The usual way is to first run a model assuming no heterogeneity and then build alternative models with a prespecified number of latent classes. The first model can be used to feed suitable parameters and guide subsequent models with multiple latent classes. The model with the optimal number of latent classes is determined by selection metrics such as BIC or AIC [39]. The regression estimate of response variables, or coefficient, can be interpreted as increasing or decreasing the probability of membership for each specified class [38]. We followed the same standard practice in this analysis by sequentially defining the classes, beginning with no heterogeneity to 5 groups. We used the *lcmm* package for modeling the MMSE trajectory, and BIC was used to choose the optimal number of latent subgroups [40]. We then went ahead with computing model estimates for the best identified latent subclass model. To achieve this, we used the *hlme* function along with the *gridsearch* option in the lcmm package [40]. We first tested a homogenous model with a single trajectory by specifying rs6859 as a covariate and random effects for measurement time without the gridsearch. Further, more complex heterogeneous models were specified individually with a consecutively increasing number of classes from two to five using the gridsearch option with 100 repetitions. The random initial values for the heterogeneous models were supplied from the homogeneous model. This was done to ensure better model convergence as the model parameters were not known a priori. The model with optimal classes was selected by comparing their BIC. We then estimated the rs6859-specific effect for the latent trajectories by adjusting for appropriate confounders. For this model, age, gender, education, smoking, measurement visits and race were adjusted as covariates along with SNP rs6859, both as fixed effects and as mixture term. Similar to the mixed models, age and education, were scaled and centred to harmonize the effect estimates. The slope was allowed to vary for the measurement visits simultaneously, accounting for subject-specific intercepts in the random effects.

## Results

### Sample characteristics

The final sample contained the records of 1312 individuals (Supplementary Figure S1), and complete covariate information was available for 1310 individuals, of which 268 were diagnosed with AD. The median number of MMSE measurements and intervals between adjacent MMSE measurements was 4 and 197 days (Supplementary Figure S2 and Supplementary Table S1) respectively. Supplementary Table S1 shows the baseline characteristics of the analyzed sample. The mean participant age was 73.74 years. There was an overrepresentation of males (55.19%), and the majority of samples where whites (92.9%). The mean duration of the education of individuals was close to 16 years. At baseline, 390 (29.77%) and 55 (4.19%) had smoking and alcohol histories respectively. At baseline, about 11% of people had diabetes, and over 40% had metabolic risk factors. The distributions for the MMSE and normalized MMSE are shown in Supplementary Figure S3. The median MMSE was 25 (IQR: 21–28). The clinically measured MMSE had a right-skewed distribution with peak values between 25 and 30. On the other hand, a more bell-shaped distribution was observed for the normalized MMSE phenotype. Above 50% of the individuals had the GA genotype for the SNP rs6859. The curvilinear relationship between the normalized MMSE and the untransformed MMSE is illustrated using a scatterplot in Supplementary Figure S3.

### Relationship of SNP rs6859 with lowest participant MMSE

The model-averaged coefficients from the linear regression model are summarized in Table 1. An increase in the A risk allele for SNP rs6859 was associated with a decrease in normalized MMSE (-2.23, 95% CI: -3.83, -0.62, *p* < 0.01). This estimated coefficient was independent of the impact of main confounders such as age, education, gender, diabetes, hypertension, and CVD. As expected, age was a risk factor for decreased cognition (-0.34, 95% CI: -0.51, -0.18, *p* < 0.001). Males were more likely to have a lower MMSE reading, as evidenced by a statistically significant regression coefficient of (-4.46, 95% CI: -6.81, -2.10, *p* < 0.001). A greater number of educational years had a protective association on the MMSE (1.77, 95% CI:1.37, 2.18, *p* < 0.001). The marriage status had no significant association with the MMSE values. Among the behavioral risk factors explored, only smoking was associated with MMSE: regression coefficient (-4.60, 95% CI: -7.11, -2.09, *p* < 0.001). As regards to the percentage of variance predicted, the contribution of rs6859, age, education, smoking, marriage, sex, diabetes status in the cross-sectional analysis respectively were 0.69, 2.05, 4.86, 1.19, 0.28, 0.86, 0.32. The estimated E-values for the univariate and multivariate models were 1.47 and 1.43, respectively. The E-values are expressed as odds ratios that were computed from the mean difference of the outcome between the carriers vs. non-carriers of rs6859 calculated from the ordinary least squares (OLS) by hypothetically considering the MMSE as a dichotomous variable [34]. Therefore, the estimated E-values suggest that the effect size of the confounder should be fairly large to rule out the rs6859 association with the MMSE.

**Table 1:** Model averaged coefficients estimated to identify the effect size of rs6859 and other covariates in the multivariate linear regression model (*n* = 1310)

Variable	Coefficient	95% CI	*p*
Age (Years)	-0.34	-0.51, -0.18	< 0.001***
Education (Years)	1.77	1.37, 2.18	< 0.001***
Gender (Male)	-4.46	-6.81, -2.10	< 0.001***
Marriage (Never)	4.36	-0.73, 11.30	0.08
rs6859 (A allele)	-2.23	-3.83, -0.62	< 0.01**
Smoking (Yes)	-4.60	-7.11, -2.09	< 0.001***
Race (White)	0.16	-3.15, 5.76	0.56
Alcohol (Yes)	0.05	-5.20, 6.27	0.85
Hypertension (Yes)	-0.001	-2.38, 2.35	0.98
Diabetes (Yes)	4.05	0.37, 7.73	< 0.05*
CVD (Yes)	0.01	-2.23, 2.47	0.92
Dyslipidemia (Yes)	0.02	-2.11, 2.55	0.85

Note. ^*^p < 0.05; ^**^p < 0.01; ^***^p < 0.001 Note. Hypertension, CVD, diabetes and dyslipidemia identified from the medication data

### Longitudinal association of SNP rs6859 with MMSE

**Fig. 1:**
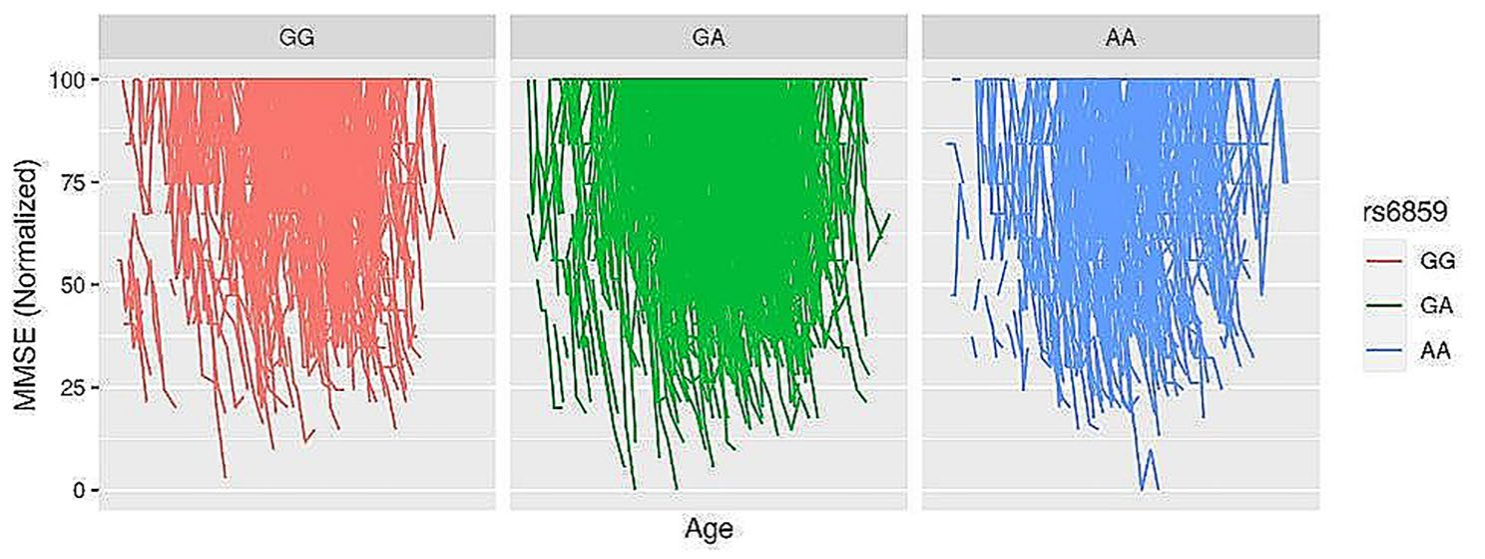
Longitudinal measures of individual MMSE in the ADNI cohort for clinical visits stratified by rs6859 genotype

**Fig. 2:**
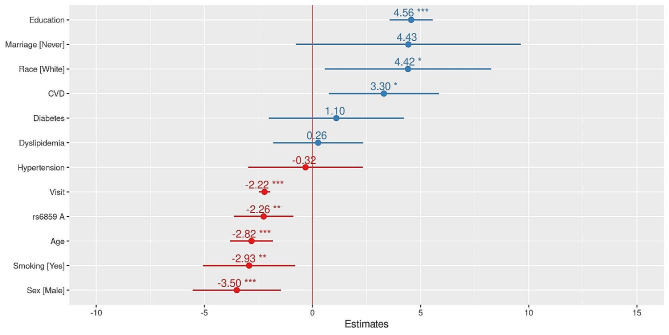
Forest plot showing the result of the covariate adjusted linear mixed models for the normalized MMSE response. Note. Clinical conditions such as diabetes, hypertension, dyslipidemia, and cardiovascular disease (CVD) were discerned from the medication files, indirectly reflecting the impact of relevant medications on the longitudinal MMSE.

**Fig. 3:**
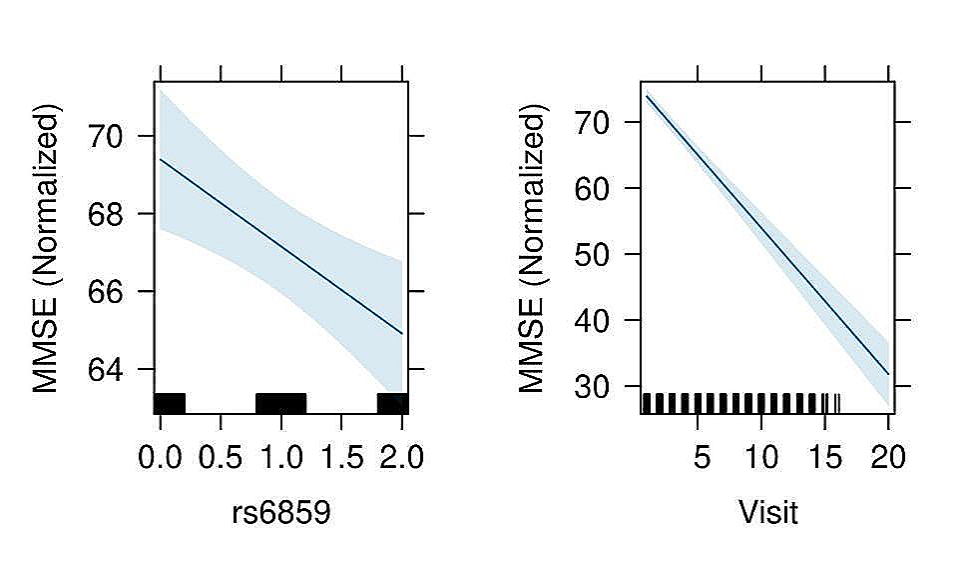
Effect plots depicting the association between rs6859 and clinical visits with normalized MMSE are represented by Figure (a) and Figure (b) respectively. Note. The plot was generated from a covariate adjusted linear mixed model. The dark blue line and surrounding light blue colour show the relationship and associated 95% confidence intervals. The black colour lines in the x-axis for figures show the density of the observed values

**Fig. 4:**
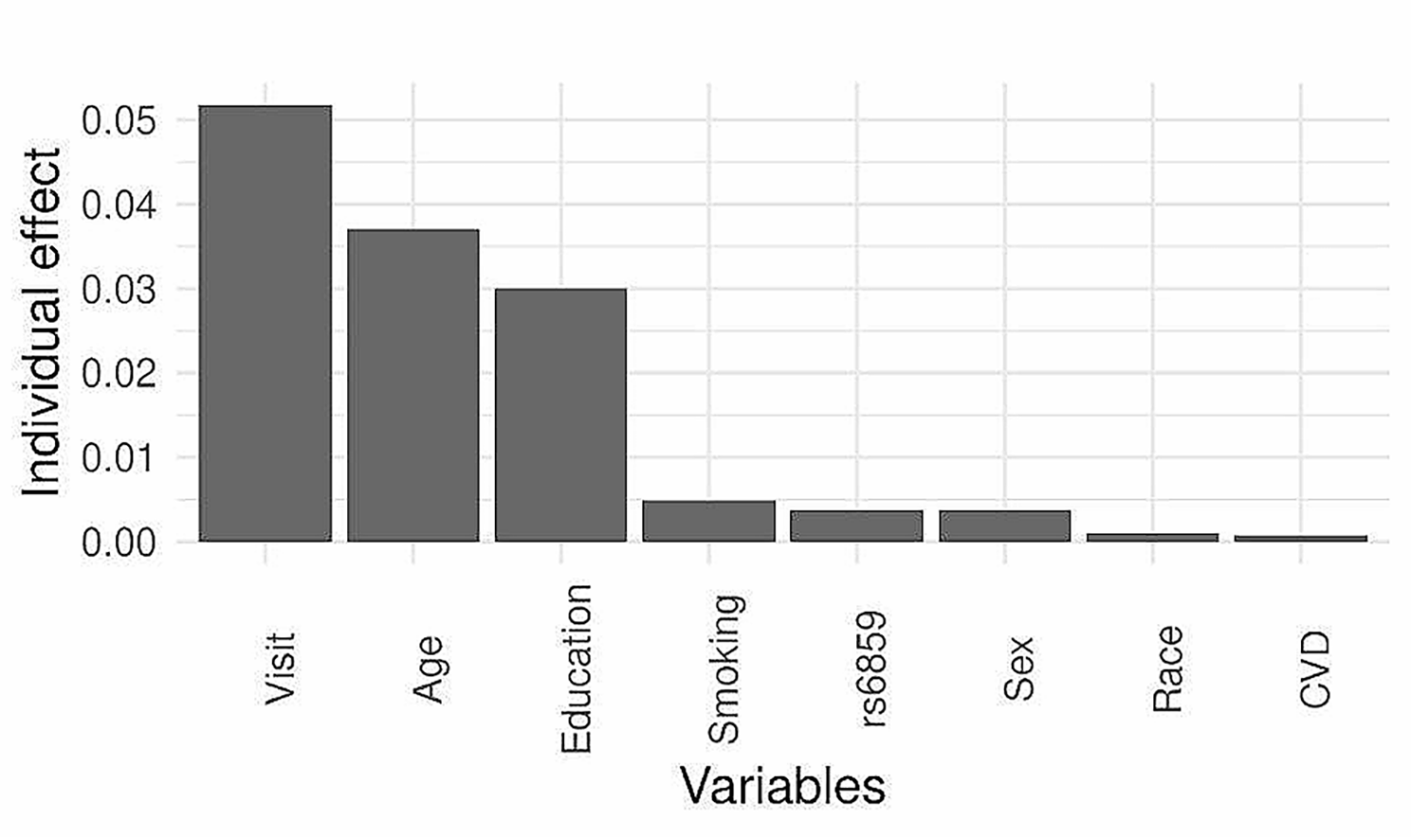
Individual variable contribution towards linear mixed model variance identified using hierarchical partitioning

The Spaghetti plot shows large individual variation in normalized MMSE with age for rs6859 genotype strata in the ADNI sample (Fig. 1). Supplementary Table S2 presents the AIC and deviance for the different models to determine the best combination of variables. The most informative model consists of rs6859, age, sex, race, education, visits, smoking, marriage, CVD, hypertension, diabetes dyslipidemia, and random effects with varying effects for visits and participants (AIC: 55,854, *p* < 0.01). The result of the mixed model analysis is presented as a coefficient plot (Fig. 2). The MMSE declined with an increase in the A allele for rs6859, which was associated with a -2.26 decline in MMSE (*p* < 0.01). Smoking status was associated with a reduced MMSE of -2.93 (*p* < 0.01). Interestingly, among the medications for clinical risk factors, CVD medication use was associated with a healthy trajectory i.e. 3.30 increase in normalized Mini-Mental State Examination (MMSE) scores. Increased duration of education and white race were associated with an improvement in the MMSE. Never-married status was associated with a better MMSE trajectory. Alcohol status and gender variables did not add any additional predictive information to the mixed model. None of the clinical variables were statistically significant in the mixed models. The effect plots visualizing association of visits and rs6859 carriers with cognitive decline are shown in Fig. 3. The total marginal *R*^*2*^ corresponding to the fixed effects of the model covariates was 0.13. Meanwhile, the conditional *R*^*2*^ representing the contribution to the total variance from both the fixed and random effects was 0.84. Individual effects of the significant set of variables from the reduced model were visualized in Fig. 4, which is the average shared effect of the variable plus its unique effect [37]. Individual contribution percentages of rs6859, age, race, sex, education, smoking, measurement visits, and CVD extracted from the parsimonious model were 2.85, 27.83, 0.75, 2.85, 22.58, 3.68, 38.86, and 0.60 respectively.

### Latent trajectory analysis and predicted class membership associations with SNP rs6859

We provide the details of model fit statistics extracted using the *gridsearch* function to identify the optimal set of subgroups given the data in Table 2. The estimates from the gridsearch support the results from Model 3, which assumes three latent classes. The respective BIC generated from the model was 56157.25 which was substantially less than the next best model assuming 4 latent classes; Model 4 (BIC − 56179.01). Class-wise distribution of posterior probabilities from the latent class regression model is provided in the Supplementary Figure S4. According to the Model 3 posterior classification, the frequency of participants in class 1, class 2 and class 3 was 559 (42.67%), 281 (21.45%), and 470 (35.88%), respectively. The addition of the clinical visits and varying subject-specific intercepts made a marked difference in the visual fit of the model and posterior classification probability. For the class 3 multivariate latent class regression model, the computed maximum log-likelihood and BIC estimates were − 28222.44 and 56617.14 respectively. R codes used for the multivariate latent class regression are provided in the Supplementary File.

**Table 2:** Estimates from grid search for identifying the optimal number of latent subgroups corresponding to the MMSE trajectories in the ADNI (*n* = 1310, observations = 6864)

Model	Subgroup number	BIC	Log Likelihood	Class 1 (%)	Class 2 (%)	Class 3 (%)	Class 4 (%)	Class 5 (%)
Model 1	1	56423.77	-28190.35	100.00				
Model 2	2	56179.07	-28053.64	37.56	62.44			
**Model 3**	**3**	**56157.25**	**-28028.38**	**41.45**	**21.52**	**37.03**		
Model 4	4	56179.01	-28024.91	24.66	20.38	36.18	18.78	
Model 5	5	56194.52	-28018.30	23.89	19.09	5.50	27.63	23.89

Note. Models were fitted with an increasing number of classes in ascending order (1–5 classes). BIC: Bayesian Information Criterion. The bold font represents the estimates of the best subclass model determined by the lowest BIC. Posterior class probabilities for belonging to each class for the respective models are also provided

**Fig. 5:**
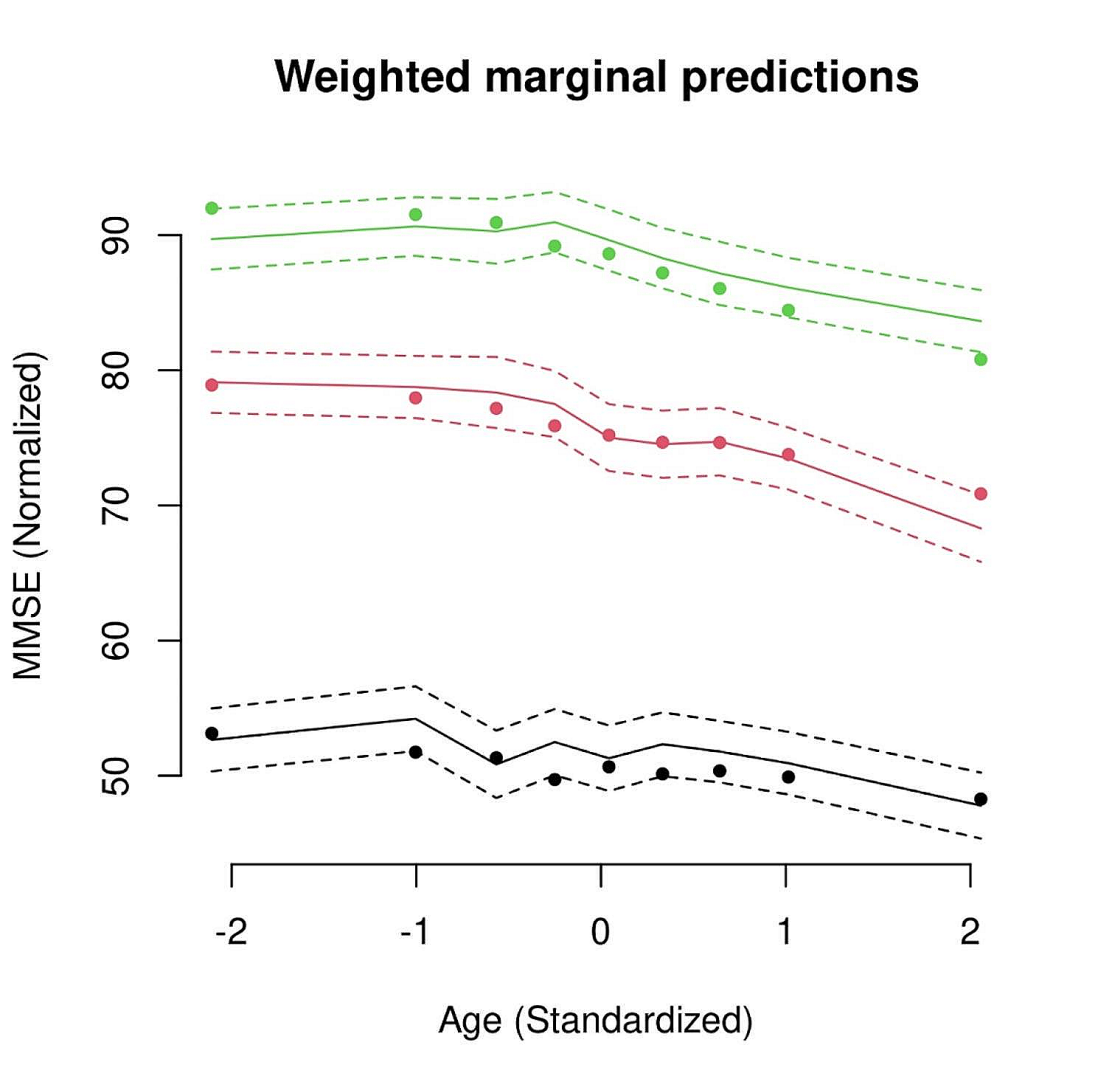
Weighted mean marginal predictions showing the trajectory of MMSE for the 3 latent classes identified from the grid search across time (*n* = 1310, observations = 6864). Note. The green, red, and black dots represent observations corresponding to classes 1, 2, and 3 respectively. The solid and dashed lines represent model-fitted lines and confidence intervals for the three latent subclasses. The x-axis shows the order of MMSE measurements from the baseline visit. The class membership probabilities were used as weights to generate the mean trajectories [40].

Figure 5 displays the characteristics of the specific classes of normalized MMSE trajectories across standardized age. There was no overlap between the identified trajectories. When analyzing the figure, the class 1 and class 2 subgroup have a vastly different trajectory to that of class 3. The common pattern was that all trajectories declined at higher ages, although the age at which decline occurred was not uniform across the classes. For all the subclasses, the baseline values determined the future trajectory. Surprisingly, we observed that the individuals in the class 1 group had the highest MMSE and maintained a relatively stable trajectory until a certain age, after which there was a gradual trend of decline. Class 2 has a lower baseline value than 80 and declines more quickly as people get older. The class 3 subgroup had a different trajectory profile compared to the rest. This group, in particular, had a much lower baseline. Individuals in this class had a MMSE (close to 52 in the normalized scale) at the beginning and were characterized by a more irregular, fluctuating trajectory. The posterior probabilities above the 0.7 threshold for the classes 1, 2, and 3 were 92.31%, 35.59%, and 73.40%, respectively. Therefore, we could conclude that the classification is satisfactory for classes 1 and 3 and there is scope for model improvement for the class 2. The characteristics of the classes 1, 2 and 3 could be summarized as stable and gradual decline, intermediate and late decline, and lowest and irregular, respectively.

Table 3 shows the regression estimates from the latent class mixed model. The SNP rs6859 could discriminate between the class 1 and class unobserved trajectories of the MMSE subgroups. The latent class regression model identified a negative slope for the classes 1 and 2; class 1 (-2.28, 95% CI: -4.05, -0.50, *p* < 0.05), class 2 (-5.56, 95% CI: -9.61, -1.51, *p* < 0.01). However, the regression coefficient pertaining to the increase in A alleles of the SNP rs6859 for class 3 was not statistically significant class 3 (-0.37, 95% CI: -1.62, 0.87, *p* = 0.55). Age and smoking status were not significant predictors of class memberships. Males were more likely to register a cognitive decline (-2.92, 95% CI: -4.37, -1.47, *p* < 0.001). An increasing number of visits was associated with a reduced MMSE trend (*p* < 0.001). Higher education was protective of long-term cognitive decline (2.79, 95% CI: 2.09, 3.49, *p* < 0.001). Diabetes, hypertension, dyslipidemia, and CVD medications were not found to be significant predictors of MMSE trajectories in the latent class analysis.

**Table 3:** Results from the multivariate latent class mixed models showing the association of rs6859 and other predictors on the trajectory of the identified 3 latent groups (*n* = 1310, observations = 6864)

Variable	Coefficient	95% CI	*p*-value
rs6859 (class1)	-2.28	-4.05, -0.50	< 0.05^*^
rs6859 (class2)	-5.56	-9.61, -1.51	< 0.01^**^
rs6859 (class3)	-0.37	-1.62, 0.87	0.55
Age	-0.42	-1.21, 0.36	0.29
Gender (Male)	-2.92	-4.37, -1.47	< 0.001^***^
Education	2.79	2.09, 3.49	< 0.001^***^
Race (White)	4.16	1.25, 7.08	< 0.01**
Visit	-1.62	-1.78, -1.46	< 0.001^***^
Smoking (Yes)	-0.83	-2.35, 0.69	0.28
Dyslipidemia (Yes)	0.61	-0.88, 2.12	0.42
Hypertension (Yes)	-0.23	-2.13, 1.65	0.80
Diabetes (Yes)	1.48	-0.92, 3.89	0.22
CVD (Yes)	1.05	-0.79, 2.91	0.26

Note. ^*^p < 0.05; ^**^p < 0.01; ^***^p < 0.001

## Discussion

Our study demonstrated that SNP rs6859 in *NECTIN2* gene, a genetic risk factor for AD [19], is associated with variation in modeling MMSE trajectory. Risk allele (A) of this SNP was associated with a statistically significant reduction in MMSE. To our knowledge, this is the first study supporting the connection between rs6859 and MMSE trajectory. Though biological functions of *NECTIN2* are still not fully understood, there is evidence that this gene can play roles in vulnerability to infections, progression of mild cognitive impairment to AD, and the formation of synapses [19, 21, 41, 42], which may contribute to its effects on MMSE.

The cognitive scales are quite dynamic and context-specific. Therefore, a single reading is less likely to mirror the underlying cognitive status [43]. MMSE was previously assessed to perform well concerning its reliability and validity credentials [44]. Scientific evidence has shown that MMSE can also connect cognitive impairment to neurodegenerative changes seen in dementia [45]. Strikingly, in mixed models, the model contribution of rs6859 was more than half due to smoking and exceeded that of marriage and race. The use of visits was shown to predict the highest amount of variance in our linear mixed model analysis. The possible explanation is that it correlates with patient characteristics and carries much more latent information than age itself. Visits are also a proxy variable for observation time. It was previously suggested that patient characteristics play an important role in determining the observation time and clinical response [46]. We found that the incorporation of the visit variable substantially improved the model fit and posterior classification in the latent class models. Age and education were adjusted as continuous variables. Morris and the team specifically warn against using them as categorical variables in regression models [47]. In keeping with prior findings, the MMSE was significantly associated with age, education, smoking, and race [6, 48, 49]. We observed that the MMSE decline rate was lower in the female and non-married groups, which runs counter-intuitive to the previous studies [50, 51]. The reasons could be that 97.9% of the non-married individuals had higher education (above 10 years) and were less frequent in our data. Importantly, carriers of *APOE4* risk alleles were more among males (350 versus 281) and less among the never married group (16 versus 615). Our analysis was limited to a subset of genotyped participants. Also, as previously noted, the characteristics of the ADNI participants may differ from those of the general population in some respects [52]. 

Blood pressure, diabetes, dyslipidemia, and cardiovascular disease (CVD) have been strongly linked to cognitive decline [53–55]. Additionally, the *NECTIN2* gene has shown considerable pleiotropic effects, influencing these phenotypes [56, 57]. Individuals who were on antiglycemic drugs at baseline were more likely to exhibit a positive MMSE trajectory in our cross-sectional analysis. Although there is some evidence suggesting a protective impact of diabetes medication on cognition [58], this could also be a chance finding as the increased number of *APOE4* alleles in the non-diabetes group and the relatively small size of the group with diabetes may both contribute to the observed protective findings. Scientific evidence indicates that splice variants within the *NECTIN2* can significantly influence the well-being of the nervous system, endothelial cells, and myocytes [59]. In mixed models, among the medication use variables, only the use of CVD medication was found to be significant and was associated with an elevated MMSE score. However, this effect did not persist in the latent class model. We are uncertain about the factors that may have contributed to the aforementioned finding. Overall, the evidence for this is not quite conclusive and need further support from longitudinal studies for confirmation [60–62]. 

Previous research demonstrated that the subclasses do not follow the trajectory predicted by linear mixed models [63]. Several studies have previously attempted to model trajectories for cognitive decline both in the general population as well as in individuals with varying levels of dementia [64–67]. However, because the paths were so dissimilar across studies, the results could not be generalized to other investigations [66, 68, 69]. The main issue was the inconsistency in the number and characteristics of trajectories due to differing populations, the degree of cognitive illness of the participants, and the heterogeneous effects of covariates across latent classes [66, 69, 70]. It was noted in a systematic review that the latent class algorithms tend to frequently choose between three to four classes with varying MMSE slopes, which were in turn correlated with the MMSE baseline readings [71]. Moustafa et al. had earlier shown that a linear link function fits the ADNI MMSE data well. However, unlike our study, the mean MMSE trajectory changes were measured separately for the CN, MCI, and AD groups. While there were no observed MMSE changes for CN, there was a decline in MMSE trajectories for the other two groups [72]. In our case, we used more MMSE readings, and therefore the latent classes were more likely to experience a cognitive decline. We found that SNP rs6859 was significantly associated with two of the three identified classes of heterogeneous MMSE trajectories. Furthermore, we demonstrate that while the mean response modelled in mixed models is informative, it does not entirely reflect the heterogeneity in MMSE decline. For example, carriers of rs6859 polymorphisms experienced a profound rate of MMSE decline in Class 2 which was more than double the estimate from mixed models. Even though our approach was agnostic to the clinical diagnosis of cognitive status, all the trajectories were separated and showed a declining trend with age.

There are still large gaps in the scientific literature to be considered before we can make meaningful connections between infections and AD. There were inconsistent results between the discovery and validation cohorts for the causal relation between AD and common infections [73]. The *NECTIN2* gene entails a glycoprotein that is involved in the maintenance of tissue integrity and has a wider expression pattern across tissues (https://www.ncbi.nlm.nih.gov/gene/5819#gene-expression). Nectins are actively involved in the development of synapses [74]. A cross-species study had shown that Nectin molecules in general were evolutionally conserved [75]. Along with *TOMM40* and *APOE4* on the same chromosome, *NECTIN2* has been shown to be involved in longevity and dementia [76, 77]. Carriers of A allele (GA and AA genotypes) of rs6859 also have a greater rate of conversion from mild cognitive impairment (MCI) to AD than non-carriers [41]. Intriguingly, the same study found that higher concentrations of low-density lipids (LDL-c) in the carrier group attenuated this risk by almost 50%.

An additional strength of this study is that is found significant associations of rs6859 with MMSE both cross-sectionally and longitudinally. We used E-values for sensitivity analysis and model diagnostic methods such as posterior classification to ensure the quality of classification. Additionally, latent class models can accurately model the time-dependent effects of covariates even when there are measurement imbalances [78]. We also controlled for the impact of education, smoking, age, gender, and race in our work. Our analysis also generated a novel finding that SNP rs6859 is an independent predictor of inter-individual MMSE changes. The availability of genetic data and clinically measured series of MMSE measurements in the same database was another notable strength.

A major limitation of this study was that MMSE measurements in the data were gathered after 55 years. We are, therefore, unsure of the applicability of the SNP associations to comparatively younger ages. The limited representation of the non-White groups may pose issues for the external validity of our findings. The inclusion of more predictors such as physical activity, BMI, and environmental exposures may help improve the model fit [79–81]. We plan to investigate the association of rs6859 with other brain phenotypes in the context of dementia in future research.

## Conclusions

This study found statistical evidence supporting the classification of three latent subclass groups representing complex MMSE trajectories in the ADNI cohort. The SNP rs6859 in *NECTIN2* gene can be suggested as a candidate genetic predictor of variation in modeling MMSE trajectory, as well as for identifying latent classes with higher baseline MMSE. Functional studies may help further elucidate this relationship. It is also important to further explore direct pathological links between rs6859 and cognitive changes.

### Electronic supplementary material

Below is the link to the electronic supplementary material.

Supplementary Material 1

## Data Availability

Following the ADNI consortium’s approval of the request, ADNI datasets are made available to the public (http://adni.loni.usc.edu).

## Abbreviations

AD: Alzheimer’s Disease
ADNI: Alzheimer’s Disease Neuroimaging Initiative
AIC: Akaike Information Criterion
APOE: Apolipoprotein E
BIC: Bayesian Information Criterion
CN: Cognitively Normal
CSF: Cerebrospinal Fluid
GWAS: Genome-wide association studies
IQR: Interquartile Range
LCMM: Latent class mixed models
MCI: Mild Cognitive Impairment
MMSE: Mini-Mental State Examination
SD: Standard Deviation
SNP: Single Nucleotide Polymorphism
NECTIN2: Nectin Cell Adhesion Molecule 2 (gene)
