# Supplementary material for: The SNP rs6859 in NECTIN2 gene is associated with underlying heterogeneous trajectories of cognitive changes in older adults
Source: BMC Neurol. 2024 Feb 27;24:78. doi: 10.1186/s12883-024-03577-4 (PMC10898142; doi:10.1186/s12883-024-03577-4)
Supplement: Supplementary file 1 — Supplementary Material 1 [file 12883_2024_3577_MOESM1_ESM.docx]

**Supplementary Materials**

**Title: The SNP rs6859 in *NECTIN2* gene is associated with underlying heterogenous trajectories of cognitive changes in older adults**

Aravind Lathika Rajendrakumar^1^, Konstantin G. Arbeev^¶^, Olivia Bagley^1^, Anatoliy I. Yashin^1^, Svetlana Ukraintseva^1^, for the Alzheimer’s Disease Neuroimaging Initiative^§^


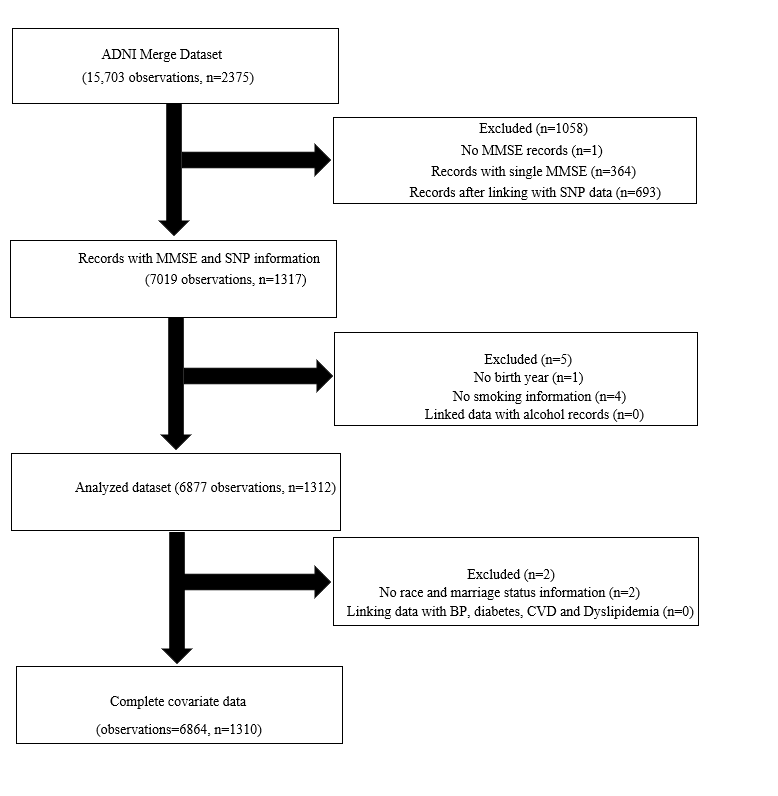


**Supplementary Figure S1.** Flow chart showing the sample selection for the association analysis


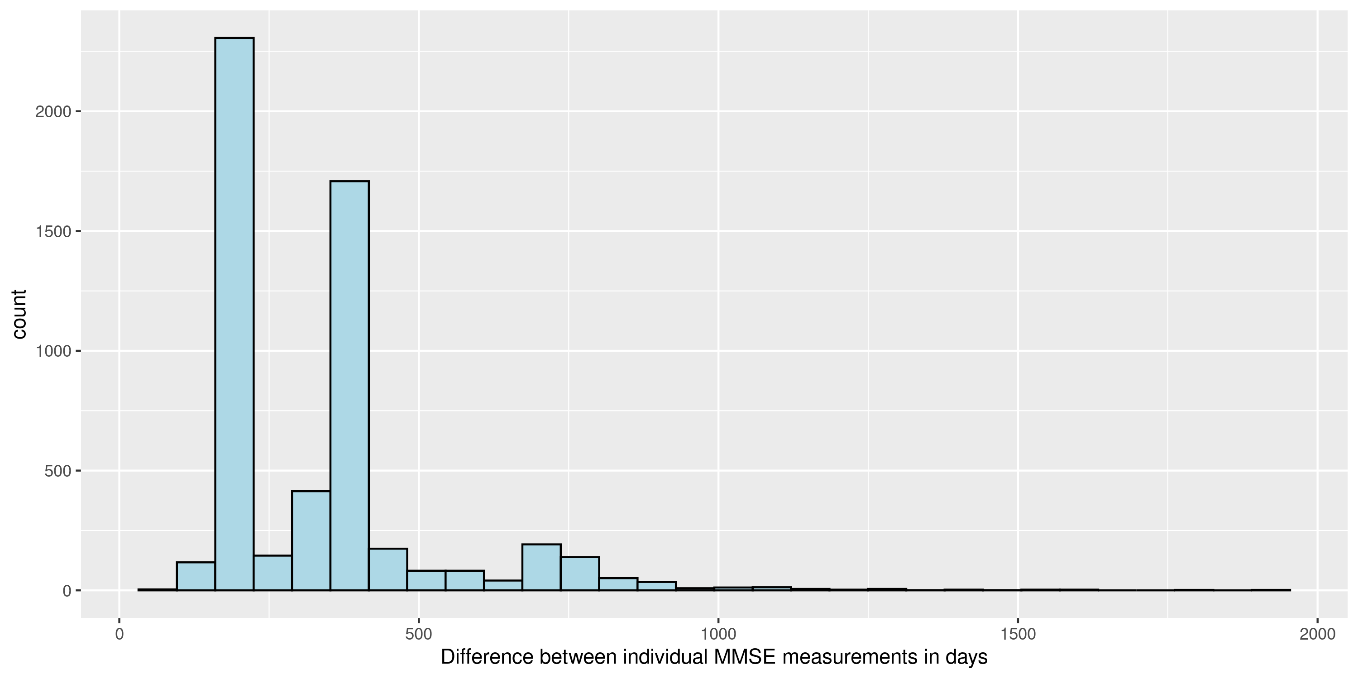


**Supplementary Figure S2.** Histogram showing the interval between adjacent MMSE measurements in the analyzed sample (observations=6864)


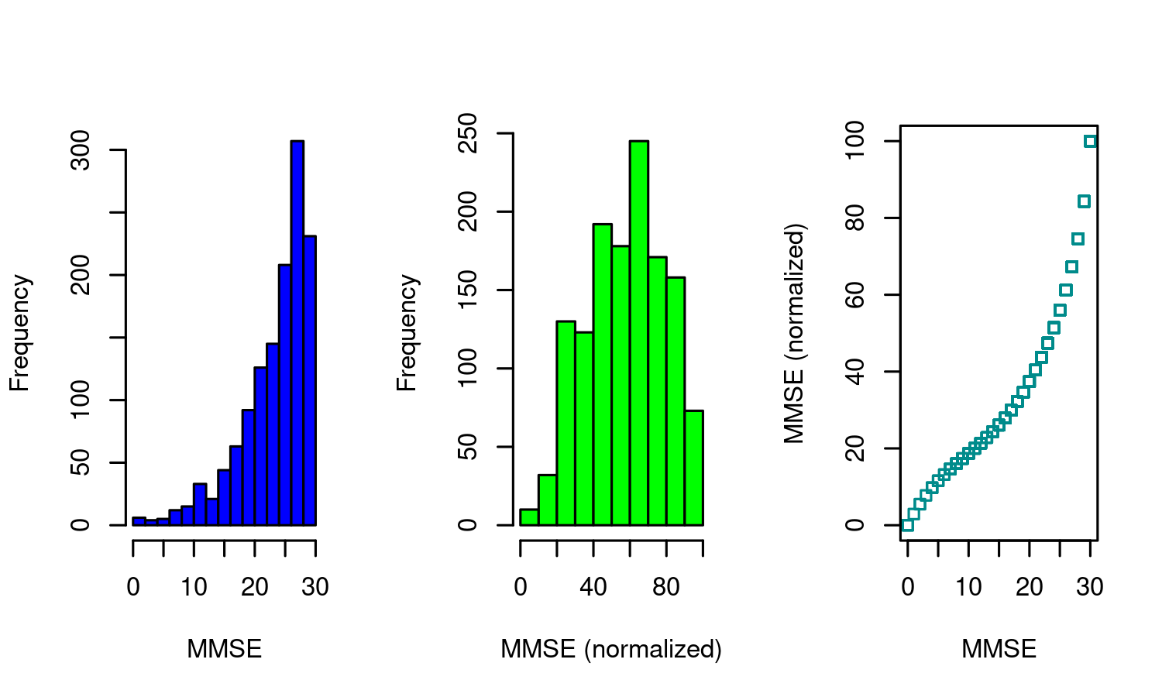


**Supplementary** **Figure S3.** Distribution of MMSE and normalized MMSE and their relationship in the analyzed sample in the cross-sectional analysis


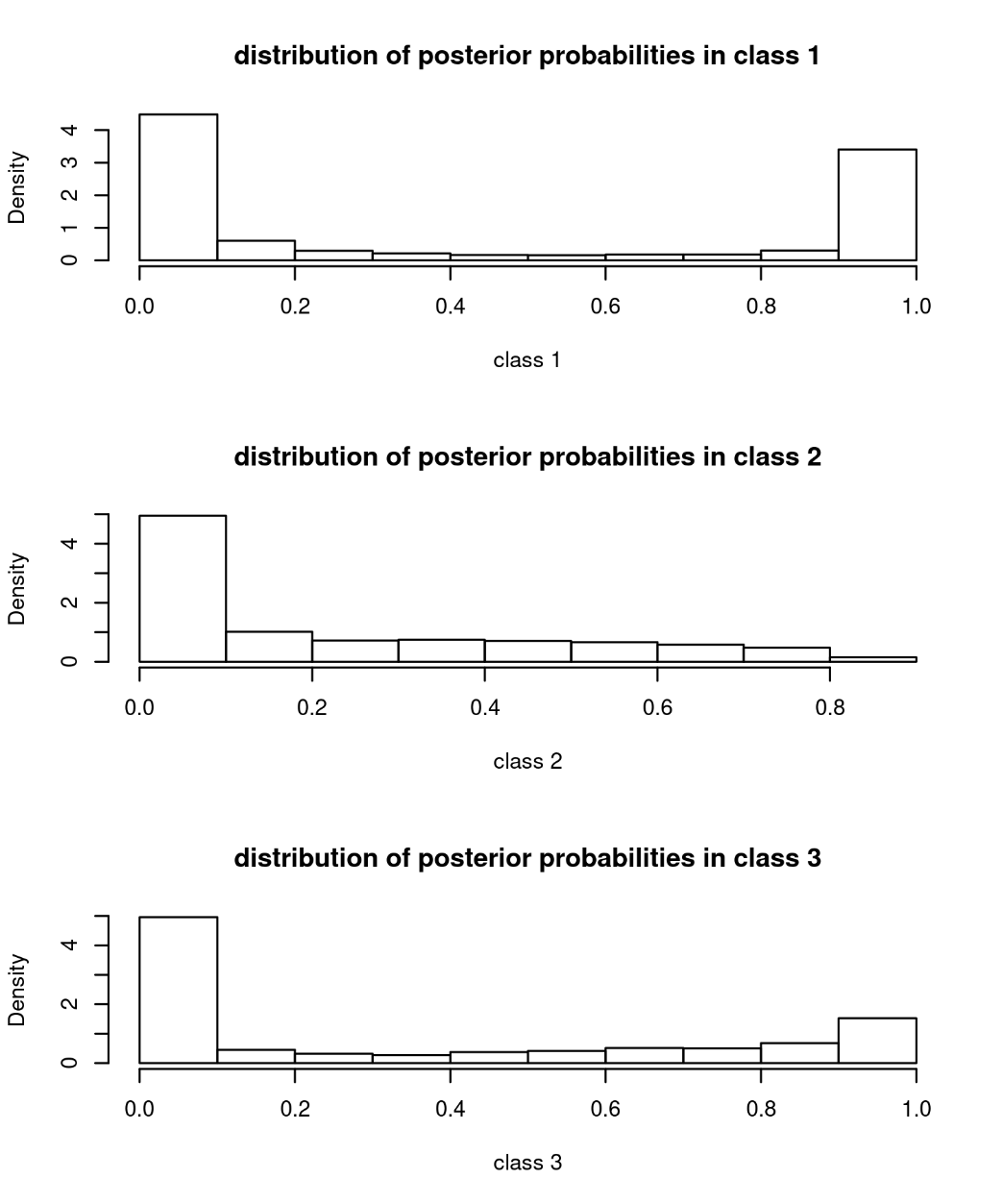


**Supplementary** **Figure S4.** Class wise distribution of posterior probabilities from the latent class regression model

**Supplementary** **Table S1.** Participant characteristics in the analyzed sample from the ADNI cohort in the cross-sectional analysis

| **Characteristics** | **Mean (Frequency)** | **SD** | **Range** |
| --- | --- | --- | --- |
| Age, years | 73.74 | 7.00 | 55.00 - 90.90 |
| Sex, Male, n (%) | 723 (55.19%) |  |  |
| Education, years | 15.97 | 2.84 | 4-20 |
| MMSE^†^ | 25.00 | 21-28 | 0-30 |
| Visits^†^ | 4 | 3-6 | 2-16 |
| **Marriage Status** |  |  |  |
| Ever | 1262 (96.33%) |  |  |
| Never | 48 (3.66%) |  |  |
| **Race** |  |  |  |
| White | 1219 (93.05%) |  |  |
| Black | 54 (4.12%) |  |  |
| Asian | 21 (1.60%) |  |  |
| Others | 16 (1.22%) |  |  |
| **Smoking** |  |  |  |
| Ever | 390 (29.77%) |  |  |
| **Alcohol** |  |  |  |
| Ever | 55 (4.19%) |  |  |
| **SNP rs6859**^$$^ |  |  |  |
| GG | 347 (26.48%) |  |  |
| GA | 661 (50.45%) |  |  |
| AA | 302 (23.05%) |  |  |
| Hypertension, Yes, n (%) | 779 (59.46%) |  |  |
| Diabetes, Yes, n (%) | 140 (10.68%) |  |  |
| CVD, Yes, n (%) | 526 (40.15%) |  |  |
| Dyslipidemia, Yes, n (%) | 758 (57.86%) |  |  |

Note. Data are presented as mean ± standard deviation (SD) or percentage (%) for continuous and categorical variables, respectively; ^†^MMSE and Visits was presented as median and IQR due to skewed distribution. ^$$^Allele frequencies of SNP rs6859 alleles in the sample.

**Supplementary** **Table S2.** Model selection parameters corresponding to the linear mixed models tested to determine the most informative model

| **Model** | **AIC** | **Deviance** | **p** |
| --- | --- | --- | --- |
| Model 1 | 55939 | 55909 |  |
| Model 2 | 55860 | 55830 |  |
| Model 3 | 55854 | 55820 | <0.01** |

Note. Model 1 adjusted for rs6859, age, sex, visits smoking, alcohol, hypertension, diabetes, CVD, dyslipidemia, and random effects (1+visit | participant ID). Model 2 adjusted for rs6859, age, sex, race, education, visits, hypertension, diabetes, CVD, dyslipidemia, and random effects (1+visit | participant ID). Model 3 adjusted for rs6859, age, sex, race, education, visits, smoking, marriage, hypertension, diabetes, CVD, dyslipidemia, and random effects (1+visit | participant ID). ^**^p<0.01.

R code used for deriving model parameters from a homogenous mixed model and estimating covariate effects for the optimal 3 latent class model

**## for replicability**

set.seed (1234)

**## homogenous mixed model**

train=hlme(MMSE_norm~rs6959+Age+Gender+Education+Race+Visit+Smoking+Dyslipidemia_medication + Hypertension_medication +Diabetes_medication + CVD_medication, random=1+Visit | RID, subject= “RID”, data=mmse_valsorted_ordered)

**## run the latent 3 class mixed model regression**

test=hlme(MMSE_norm~rs6959+Age+Gender+Education+Race+Visit+Smoking+ Dyslipidemia_medication + Hypertension_medication+Diabetes_medication +CVD_medication, mixture =~rs6859, subject= “RID”, ng=3, maxiter=100000, B=train, data=mmse_valsorted_ordered, nwg=T)
